# Enablers and barriers of clinical trial participation in adult patients from minority ethnic groups: a systematic review

**DOI:** 10.1186/s13063-025-08769-y

**Published:** 2025-02-22

**Authors:** Germán Andrés Alarcón Garavito, Katie Gilchrist, Coziana Ciurtin, Sanjay Khanna, Pinkie Chambers, Nick McNally, Edward Merivale, Edi Carr, Rosamund Yu, Cecilia Vindrola-Padros

**Affiliations:** 1 Rapid Research Evaluation and Appraisal Lab (RREAL), University College London, London, UK; 2 Roche Products Ltd, Welwyn Garden City, UK; 3 University College London Hospitals NHS Foundation Trust (UCLH), London, UK; 4 Public Contributor, London, UK

**Keywords:** Clinical trials, Systematic review, Barriers and facilitators, Underrepresentation, Recruitment, Inclusivity

## Abstract

**Background:**

Clinical trials are essential to the development of healthcare innovations that advance life expectancy and improve quality of life. However, there exists a pronounced disparity in ethnic representation among trial participants. This imbalance, particularly in relation to minority ethnic groups, can lead to a limited understanding of how therapies affect diverse populations. The present systematic literature review (SLR) aims to identify the factors that both hinder and facilitate the participation of minority ethnic groups in clinical trials.

**Methods:**

This review involved a systematic search of keywords across four databases: Web of Science, PubMed, CINAHL Plus and The Cochrane Library. The review was not restricted by language or study site; however, the date of publication was limited between 1st January 2017 and 1st October 2022. Studies discussing or outlining the involvement of minority ethnic groups in clinical trials, and those outlining inclusive recruitment and participation procedures were targeted.

**Results:**

A total of 43 articles were included in the review. Of these, 36 articles were from the United States (US), 20 articles reported on oncology trials and 39 articles reported information from the patient’s perspective.

Reported barriers included a lack of researchers from minority ethnic groups implementing and conducting clinical research, inadequate funding for clinical trial efforts in geographical areas serving minority populations and a lack of awareness and education among research staff regarding which underrepresented groups to target for recruitment and the strategies to employ in reaching out to them.

Several recommendations were suggested by the articles included in the review to address these barriers. Prominently, the use of patient navigators or community liaison roles was highly recommended as a way of supporting patients through the research recruitment process. The articles also highlighted the benefits of translating study materials and interventions into multiple languages and actively involving diverse communities in the development of health education materials. Lastly, leveraging technologies to address socioeconomic barriers, such as the use of virtual approaches to avoid lengthy travel, may also help to improve diversity in trials.

**Conclusions:**

Ensuring representation of minority ethnic groups in clinical trials is critical to developing therapies with generalisable efficacies. While progress has been made in enhancing outreach of wider racial groups and fortifying educational resources, there remains a pressing need to delve deeper into the obstacles impeding the recruitment of a diverse participant base, particularly in regions outside the US, where relevant studies are scarce.

**Registration:**

The review protocol was registered on PROSPERO (CRD42022368106) (1).

## Introduction

Clinical trials play an indispensable role in shaping health innovations and therapeutic interventions.

We followed the National Health Institute definition of clinical trials, as being research studies performed in people that are aimed at evaluating “a medical, surgical, or behavioural intervention”. Clinical trials may also test ways to identify disease early, or to look at how to improve the quality of life for people living with a chronic health problem [2].

Historically, there has been a lack of ethnic representation in clinical trial participants; minority ethnic groups have often been underrepresented or omitted from research [3, 4]. This exclusion persists despite some ethnic populations bearing a disproportionately high burden of certain diseases [5, 6]. The consequences of such underrepresentation are profound, leading to disparities in access to treatment and significant knowledge gaps regarding how certain drugs and therapies affect different ethnic populations [7, 8].

A frequently cited reason for the underrepresentation of certain minority ethnic groups in clinical trials is a pervasive mistrust in the healthcare system. This is often attributed to historical instances of mistreatment and exploitation. Two infamous examples of such unethical and exploitative research are often quoted: the Tuskegee Syphilis study, in which Black men were intentionally denied treatment for syphilis; and the Henrietta Lacks (HeLa) incident, where the patient’s cancer cells were provided to researchers unbeknownst to her. The subsequent research with the HeLa cell line now underpins much of the modern medical profession and practice, yet was initiated without her knowledge or consent [3, 8–13].

Such instances of mistreatment are considered to have contributed to a legacy of distrust among some ethnic minority groups. Beyond these historical issues, present-day challenges such as language barriers, cultural differences and a lack of cultural competence can make it difficult for researchers to effectively rebuild trust and establish clear communication with potential participants [14–17].

In the US, efforts to address the lack of ethnic diversity among clinical trial participants led to the implementation of the Revitalization Act in 1993. However, the success of this initiative has been mixed. A 2019 review revealed that of the cancer therapies approved by the US Food and Drug Administration (FDA) between 2008 and 2018, only 63% of the relevant trials reported race-related data [18].

Among these, only 3.1 and 6.1% of trial participants identified as Black and Hispanic, respectively. In 2022, a study by Reihl et al. revealed that, almost 30 years after the Revitalization Act was put in place in the US, White males were still overrepresented in neuro-oncology clinical trials, accounting for more than 90% of trial participants. In contrast, minority ethnic groups such as Black and African Americans, Hispanic and Latino, and Asian and Pacific Islanders each accounted for less than 2.0% of trial participants, and American Indian and Alaska Natives accounted for a mere 1.3% of trial participants [19].

Echoing these findings, similar results were seen in the 2022 UK’s National Institute for Health and Care Research (NIHR) Diversity Data Report, which published findings from 148 randomised controlled trials (RCTs) initiated between 2007 and 2017. Only 60% of these trials reported data on the ethnicity of participants. Of these participants, 86% of participants identified as White, while only 4 and 5% of participants identified as Black and Asian, respectively [20].

Yet, multiple sources indicate that in some diseases, such as cancer, the incidence and mortality rates tend to be lower among White ethnic groups in the UK compared to other groups [21–23].

A recent initiative funded by the NIHR, named “Innovations in Clinical Trial Design and Delivery for the Under-served” (INCLUDE), proposed a framework to increase the number of underserved trial participants from minority ethnic groups [24–26]. However, as the initiative only started in late 2020, the long-term impact of this framework, in genuinely increasing representation and the subsequent applicability of research findings, is yet to be determined.

While there are increasing efforts to explore the issue of inequity in clinical research participation, a holistic global review of this pressing matter does not yet exist. This may, in part, be due to the tendency among researchers to focus on specific countries, diseases and impacted communities.

The present systematic review draws together available evidence on the barriers and enablers in the recruitment and participation of minority ethnic groups in clinical trials. The aim of this review sought to determine the minority ethnic groups that face the most significant under representation, and to identify reasons for their potential exclusion from trial enrolments, reasons for declining or withdrawal, and the factors that motivate certain individuals from these groups to accept and persist in trial participation.

The findings from this review will inform the next phase of the investigation into the barriers to recruitment of ethnic minority patients to clinical trials; a qualitative study in which relevant patients and staff will be interviewed across multiple disease areas.

## Methods

A phased approach was adopted for the present review, which started with a broad search strategy, and subsequently expanded with each round of searches. The Preferred Reporting Items for Systematic Reviews and Meta-Analysis (PRISMA) statement was followed to guide the review design and the reporting of the methods and findings. A protocol was developed a priori and was registered on PROSPERO (CRD42022368106) [1].

The UK government’s definition of people from ethnic minority groups was used to classify individuals with ethnic characteristics that are different from the majority population of a region [27]. As a result, the categorisation of “minority ethnic groups” might differ between geographical locations and countries.

### Project steering group

We consulted with a Steering Group, which included patient representatives, researchers, clinicians and pharmaceutical industry leaders with expertise and/or interest in improving the access of patients from minority ethnic groups to clinical trials. Meetings took place at key stages of the review, including protocol drafting, evidence searching, and the analysis of the findings. These meetings were developed in group calls where participants were presented with preliminary ideas, updates and emerging findings.

During these sessions, some of the discussion points included perspectives around themes that participants were expecting, how useful this information would be in their practice/research, and ideas for the dissemination of findings. After each meeting, minutes were reviewed to ensure that participants’ feedback was integrated in next iterations of the review.

### Search strategy

Search terms were identified using a combination of free-text and controlled terms. The terms were assessed and refined through exploratory searches in Web of Science, PubMed, CINAHL Plus and The Cochrane Library. A provisional search strategy was tested for sensitivity versus breadth using different combinations of Boolean operators and search strings. See Appendix 1 for the complete search strategy.

In order to formulate an optimal strategy that would generate manageable yet informative findings, the search was limited to articles published between January 2017 and October 2022. This decision was also influenced by the observation that consistent terminology pertaining to health disparities has predominantly become commonplace only in recent years. Also, increasing the search window generated inconsistent and extensive results that may obscure conclusions. The search had no language or study location limitation.

The search strategy focused on three categories: trials, involvement/enrolment and minority ethnic groups. The definition for “minority ethnic groups” categorised individuals based on their ethnicity characteristics that are different from the majority population of a region [27]. Final searches were conducted in October 2022 across four databases (Web of Science, PubMed, CINAHL Plus and The Cochrane Library/ the Cochrane Central Register of Controlled Trials (CENTRAL)).

### Document selection

The search results were imported into Rayyan, a validated tool with semi-automated features enabling the detection of duplicated publications from the different databases. Rayyan facilitated the screening by presenting citation details, titles and abstracts of each publication [28].

A combined initial screening of title and abstract for eligibility was then conducted. Following the initial screening at the title and abstract level, a single researcher verified 10% of exclusions against the inclusion criteria. Subsequently, the remaining publications that met the inclusion criteria underwent full-text screening for eligibility. To ensure accuracy, 100% of included and 5% of excluded documents were checked by another reviewer, and discrepancies were resolved via discussion.

To summarise, we applied the following inclusion and exclusion criteria:

### Inclusion criteria

- Peer-reviewed papers or manuscripts where the participation or involvement in clinical trials of individuals from minority ethnic groups was mentioned and/or described
- Peer-reviewed papers or manuscripts where inclusive recruitment and participation processes were described
- Empirical studies
- Individuals (adults) identified as potential participants in clinical trials. We excluded studies focused on paediatric patients due to the added complexity of recruitment and informed consent processes for this population.
- Articles published between January 2017 and October 2022

### Exclusion criteria

- Paediatric studies.
- Grey literature.

### Data extraction

Data extraction was conducted using an extraction form on the web-based Research Electronic Data Capture (REDCap) database to organise the review process. The extraction form was first tested, and necessary amendments were made before extracting data from the included documents. The study details that were extracted from all articles included information on identifiers (authors, year of publication, location, type of article and study design), demographics, barriers and motivations to participate or be involved in clinical trials, among others.

The data extraction was completed by one researcher (GAAG), and 50% of the extracted records were independently verified by a second researcher (KG). Preliminary extractions and findings were discussed between researchers, and any discrepancies were discussed until consensus was reached.

### Data synthesis

A narrative synthesis was used to describe the overall findings noting any variations within the studies [29]. The analysis focused on developing themes that could provide an accurate representation of the included articles. The categories for the analysis were based on the research questions guiding the review as well as the information emerging from the documents.

### Quality assessment

The methodological quality of the empirical studies was critically appraised using the Mixed Methods Appraisal Tool (MMAT) [30, 31]. The MMAT was developed to allow the team to assess the methodological quality of diverse study designs, including qualitative, quantitative and mixed methods. The assessment was performed using a scale of 0 to 5, considering the number of positive or negative points on the five appraisal questions.

## Results

### Article selection

The initial search yielded 21,026 articles. After using Rayyan and Mendeley deduplication features, 2,600 papers were removed. The remaining 18,426 records were screened at the title and abstract level, and 18,156 articles were excluded as they did not meet the inclusion criteria outlined above. From the 270 records that were sought for retrieval, 7 papers were not available in full text. The remaining 263 articles were reviewed at the full-text level. However, 220 of these were subsequently excluded due to the following reasons:

1. Only researching demographics and not the recruitment process or patient experiences specifically (irrelevant analysis or characterisation)
2. Type of document
3. Unrelated
4. Wrong population
5. Not focused on clinical trials
6. Not focused on minority ethnic groups.

**Fig. 1:**
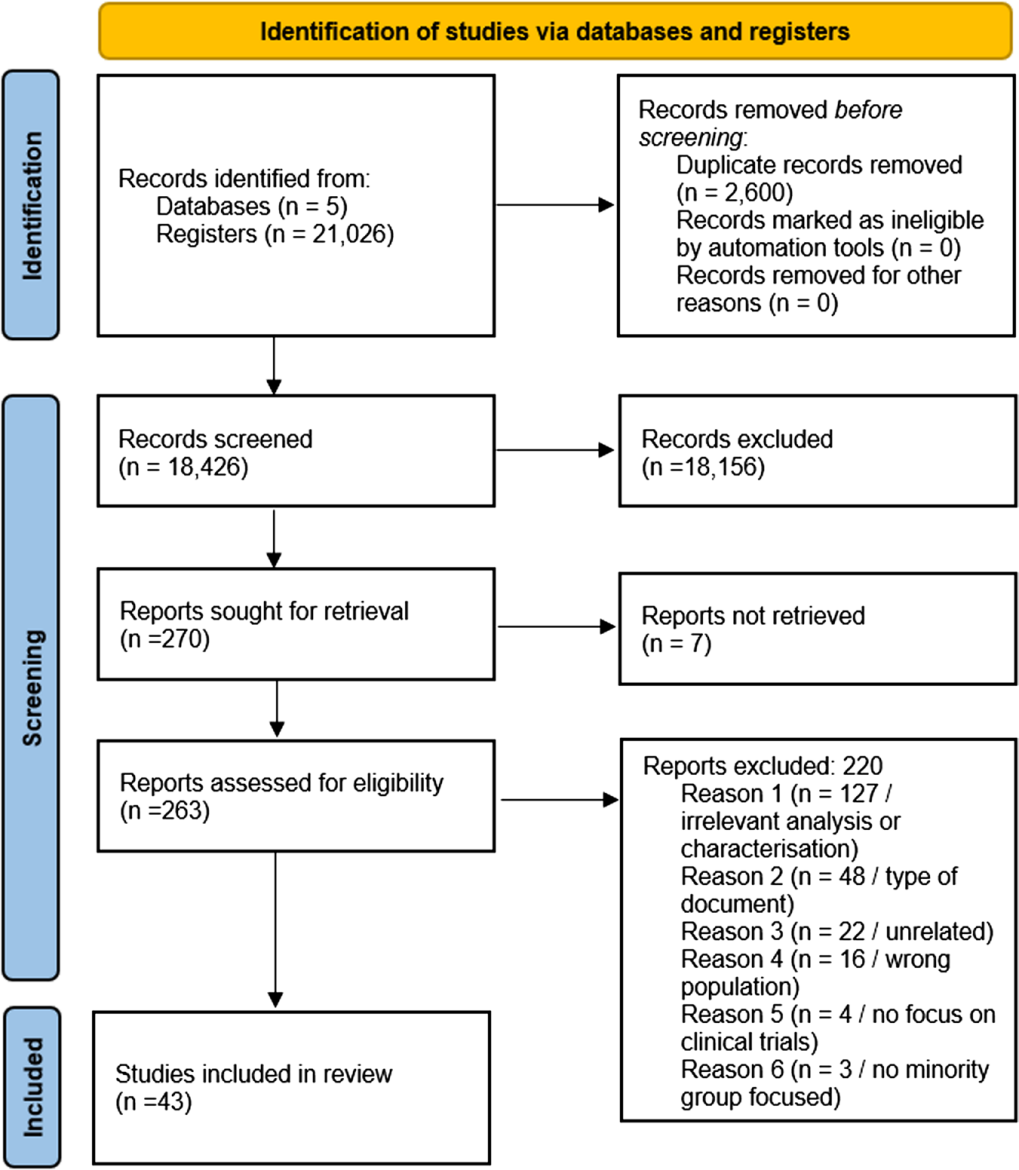
The PRISMA flow diagram

A total of 43 articles were included in the final review (See Fig. 1 for the PRISMA flow diagram).

### Article characteristics

Of the 43 articles included in the final review, 36 articles were from the US, two each from the UK and Australia, and one each from Germany, South Africa and a global perspective. Of these, 27 records used a quantitative research design, 9 were qualitative and 7 used a mixed-methods design. Although 20 articles did not specify the type of minority ethnic group, 17 focused on one group and 6 on two groups. The minority ethnic groups reported were predominantly African Americans, Black and Latin Americans or Hispanics.

In terms of clinical focus, 20 of the articles included in the review referred to oncology trials, 7 to neurology, 4 to cardiovascular, 5 to non-condition specific trials, 3 to immune diseases, two to respiratory diseases and one each to HIV and rheumatology. Of the oncology trials, 13 were not specific to cancer type; while others were type-specific, including two for haematological cancer, and one each for breast, colorectal, gynaecologic, pancreatic and prostate cancer. The neurology trials were related to Alzheimer’s disease, multiple sclerosis and stroke, while cardiovascular included heart failure and antithrombotic treatment trials.

From a narrative angle, 4 papers reported information from staff perspectives, while the majority highlighted patients’ perspectives. As for gender specificity, 36 articles were not sex-specific, 4 were focused on women and 3 focused on men. A summary of article characteristics is shown in Table 1.

**Table 1:** Article characteristics

Authors/year	Study location	Type of article	General population of interest (as reported in the source)	Disease area	Patient or staff?	MMAT score
[32]	USA	Quantitative study	Asians and Native Hawaiians	Oncology	Patients	4.5
[33]	USA	Mixed methods	African American	Cardiovascular	Patients	5.0
[34]	USA	Qualitative study	Not specified	Immune diseases	Patients	4.5
[35]	USA	Quantitative study	Not specified	Oncology	Patients	3.5
[36]	USA	Quantitative study	Hispanic	Neurology	Patients	3.5
[9]	USA	Quantitative study	African American	Neurology	Patients	2.5
[37]	USA	Quantitative study	Not specified	Oncology	Staff	3.0
[38]	Australia	Quantitative study	Indigenous	Oncology	Patients	3.0
[10]	USA	Qualitative study	African American	Oncology	Patients	5.0
[39]	USA	Quantitative study	Not specified	Oncology	Patients	4.0
[40]	USA	Quantitative study	Not specified	General	Patients	3.0
[41]	USA	Quantitative study	Not specified	Oncology	Patients	4.0
[42]	USA	Quantitative study	African American	Oncology	Patients	4.0
[43]	USA	Qualitative study	African American	Oncology	Patients	5.0
[44]	UK	Qualitative study	Not specified	Neurology	Staff	4.0
[45]	USA	Qualitative study	African American	Oncology	Patients	5.0
[46]	USA	Quantitative study	African American	Genomic	Patients	2.5
[47]	USA	Mixed methods	Korean American	General	Patients	4.0
[48]	USA	Mixed methods	African American	Oncology	Patients	4.5
[49]	USA	Quantitative study	Not specified	Respiratory diseases	Patients	4.0
[50]	USA	Quantitative study	Black and Hispanic	Oncology	Patients	3.5
[17]	USA	Mixed methods	African American	Neurology	Patients	5.0
[14]	USA	Qualitative study	Not specified	General	Patients	5.0
[16]	Germany	Quantitative study	Not specified	Cardiovascular	Patients	4.0
[51]	USA	Quantitative study	Black and Latin-American	Oncology	Patients	4.0
[52]	UK	Mixed methods	Not specified	General	Patients	4.5
[53]	USA	Mixed methods	Not specified	Oncology	Staff	5.0
[54]	USA	Qualitative study	Not specified	General	Staff	5.0
[55]	USA	Quantitative study	Not specified	Oncology	Patients	4.0
[56]	South Africa	Quantitative study	Not specified	HIV	Patients	3.5
[57]	Global	Quantitative study	African American and Hispanic	Neurology	Patients	3.0
[11]	USA	Qualitative study	African American	Oncology	Patients	1.0
[58]	USA	Quantitative study	Not specified	Rheumatology	Patients	3.0
[15]	USA	Quantitative study	Hispanic	Neurology	Patients	3.0
[59]	USA	Quantitative study	African American	Neurology	Patients	4.0
[60]	USA	Mixed methods	African American	Oncology	Patients	3.0
[61]	USA	Quantitative study	Not specified	Oncology	Patients	3.0
[62]	Australia	Quantitative study	Not specified	Oncology	Patients	4.0
[8]	USA	Qualitative study	Black	Immune diseases	Patients	5.0
[63]	USA	Quantitative study	Not specified	Respiratory diseases	Patients	2.5
[64]	USA	Quantitative study	Not specified	Cardiovascular	Patients	5.0
[12]	USA	Quantitative study	Latin-American and African American	Oncology	Patients	4.0
[66]	USA	Quantitative study	Black and Latin individuals	Immune diseases	Patients	3.0

* MMAT scores were graded between 1 and 5, being 1 the lowest and 5 the highest. Scores were calculated based on the number of positive and negative responses in the MMAT checklist

### Quality assessment

After assessing the included articles with the MMAT tool, the average score was 4. A total of 12 articles were assessed with a score of below 3. This included two RCTs [61, 66], three non-randomised studies [15, 46, 64], six quantitative descriptive studies [9, 37, 38, 40, 57, 58] and one mixed methods study [60]. A score of 3 denoted limitations using the selected methodology and interpreting the results.

### Characteristics of minority ethnic groups included in the studies

It was notable that of the studies targeting specific minority ethnic groups, African American and Black patients were the principal groups of interest. Although this may be attributed to location (83.7% of studies were from the US), other common groups in the US (e.g. Korean American, Hispanic and Latin American) were underrepresented in the selected literature studies [67]. Two studies focused on Indigenous communities, specifically in Australia [38], and in the US (Native Hawaiians) [32].

Although 7 studies centred on the recruitment of Hispanic or Latin American individuals [12, 15, 36, 50, 51, 57, 66], most of them did not differentiate between the terms Hispanic (with ancestry from a country whose primary language is Spanish, as per the Duke School of Medicine definition) and Latin American (with origins from anywhere in Latin America and the Caribbean) [68].

### Reasons why patients may not be invited to participate in clinical trials

Fourteen studies reported on why certain patients may have been overlooked for clinical trial participation [12, 17, 38, 42, 44, 45, 52–55, 59, 63, 66, 69].

Implicit bias of healthcare workers was a significant issue that has been researched. Two studies suggested that, due to implicit bias, healthcare workers were likely to make assumptions about whether a patient was an “ideal candidate”, which could lead to patients from certain groups not being invited to take part in trials [17, 42].

It was suggested that these assumptions might stem from healthcare workers’ prior experiences, a lack of confidence in referring patients to clinical trials, or perception of who would make “appropriate candidates” [53, 66].

There was also a notion among some healthcare professionals that recruiting minority ethnic participants might be more cumbersome, demanding additional effort, time and resources [52, 59]. In a 2021 study by Lincoln et al., patients who self-reported as African Americans cited a lack of awareness about research opportunities as a contributing factor for their marginal representation from trials and their community receiving no information about trials currently taking place. Although it might be expected that patients learn about relevant trials during outpatient appointments, participants expressed that they were attracted to learning of trials in an atypical way, such as through their community where information might be more accessible [17].

One analysis of a Clinical Trials Registry in Australia and New Zealand established that Aboriginal and Torres Strait Islander people had reduced opportunities to participate in clinical trials [38]. Factors contributing to this disparity included the geographical misalignment between trial locations and the residential areas of these communities, as well as the discrepancy between the cancer types studied in trials and those predominantly affecting Aboriginal and Torres Strait Islander people. Additionally, the study highlighted instances where trial criteria were ambiguously worded, leaving much to the interpretation or opinion of the investigator [38]. Lastly, many research sites were located in areas difficult for Aboriginal and Torres Strait Islander communities to access or required travel to main cities to access treatment and subsequent follow-up visits [38].

While some regulatory bodies, such as the FDA [70], have rolled out guidelines to stimulate more diverse recruitment to trials, implementing these changes is challenging. Factors such as the timeline of research projects, the need to build awareness for broadening research engagement, and the complexities in upskilling researchers [52], all mean that tangible shifts will take time.

### Reasons why patients declined participation

Twenty-four papers reported information on why patients might decline invitations to participate in clinical trials [9–11, 16, 17, 32–34, 39, 42–47, 51, 52, 57–59, 61, 63, 65, 69]. Barriers were reported across all stages of clinical trial design and delivery, and included socioeconomic challenges such as unstable employment, strict schedules, difficulties securing childcare, low income and transport deficiencies [32].

Education was found to be a relevant determinant of participation, as discussed in 20 studies [12, 34, 39–43, 45, 46, 48, 50, 51, 53–57, 59, 61, 63].

According to Niranjan et al. [53], some interviewees (i.e. principal investigators, research staff, referring clinicians) claimed that education or socioeconomic status, rather than race, impacted trial participation. One referring clinician believed individuals with lower levels of education or income were less trusting of clinical research than those from specific racial backgrounds [53].

Another member of staff noted that upper-to-middle-income individuals with higher levels of education were more informed about clinical trials, impacting their perceptions beyond racial or ethnic considerations [53]. This suggestion was also observed by Sneed et al. in 2021, where a patient indicated that more educated Black individuals might be less sceptical about research as they had more knowledge of the health care system [8].

In a study by Pimentel Maldonado et al. [57], participants who self-described as African American expressed concerns about data privacy, while participants who self-described as Hispanic reported concerns related to health insurance benefits or immigration status due to trial participation. Both groups feared not being fully informed about the trial details [57].

A lymphoma trial in the US reported that individuals declined trials because they felt participation would limit their time spent and commitments with families, work or social engagements. Other studies reported location constraints of clinical sites further dissuaded potential participants [10, 46, 69]. Three studies highlighted cases where patients believed that clinical trials were part of the regular health services, they were entitled to [10, 33, 45], and previous negative experiences of health services deterred them from participating in trials.

In general, studies looking at patients who self-described as Black and African-American tended to link disparities in trial participation to historical and structural forms of racism. Likewise, scepticism about general clinical research [10] research motives and intentions [11] were also cited as reasons for declining clinical research.

Seven articles reported that Black and African American patients attributed their mistrust to the legacy of unethical research conduct, such as the infamous Tuskegee Syphilis study, fearing exploitative research practices [11, 42, 44, 51, 59, 63, 69]. In a non-interventional study investigating barriers to screening uptake, medical mistrust was so deep-rooted that even unconventional outreach efforts, such as liaising with barbershops, were unsuccessful. One interviewed barber reasoned his mistrust in doctors by quoting the Tuskegee experiment [11].

Obstacles to African American trial participation also included reduced health literacy [43], poverty and seriousness of the medical condition [44]. Cultural norms and community identity were identified as barriers; for instance, a study by [17], discussed the “Southern” mindset and reluctance to seek medical care among African Americans in Alabama [17].

The role of healthcare professionals and their lack of training in working with different cultures were highlighted in three studies. Sneed et al. [8] noted that a lack of cultural competency among research staff could lead to mistrust. One patient participant pointed out that their relationship with medical services was marked by racial bias, leading to cultural mistrust of the service [63].

Granda-Cameron et al. [43] reported that patients felt that physicians did not allow sufficient time to address their concerns and were condescending, resulting in mistrust and further leading to their unwillingness to participate [43]. Furthermore, Lincoln et al. [17] revealed perceptions of racial bias in treatment among African Americans participants [17].

Studies also suggested that in Hispanic and Latin American communities, it was important that the whole family, not just the patient, was informed about trials. Fink et al. [42] cited cases where family opinions influenced patient decisions and willingness to participate [42]. Studies also highlighted that language barriers, primarily where English was not the first spoken language, was a major barrier for Hispanic and Latin American trial participation [69]. Meanwhile, in a German study, 8.5% of eligible patients who declined participation had a migrant background, with 20% of these patients citing a lack of German language skills as the reason for declining [16].

### Reasons why patients withdrew from clinical trials

The topic of patients withdrawing from clinical trials was scarcely discussed in the reviewed papers; only 4 studies explored this issue in African American and migrant groups [16, 34, 40, 46]. In Horowitz et al., participants indicated inadequate social support made it difficult for them to continue with trials [46]. Other reported reasons related to social support included time constraints, clashes with employment schedules, and conflicts with staff members [40].

Finally, in a German study, a correlation emerged between the trial phase and the migration status of participants. The proportion of migrant patients declined at each subsequent recruitment stage, indicating that patients with a migrant status were more likely to withdraw from trials [16].

### Motivations to accept and continue participating

The reasons driving people from minority ethnic groups who agreed to and continued to participate in clinical trials were discussed in 12 articles [10, 12, 33, 34, 43, 45, 48, 50, 56, 57, 63, 69]. Overall, participants were inspired by altruism [12, 33, 63] and a sense of helping others with a similar disease or to benefit future generations [34, 56]. Other studies reported that patients’ initial contact with research staff was crucial. A positive encounter cultivated or renovated trust in healthcare and research, thereby motivating patients to participate in a trial [10, 43, 45].

In Kenerson et al., participants noted their decision to consent to participate was influenced by the extent to which the research aligned with their personal values [48]. For others, the feeling of being informed and respected in the decision-making process influenced their decision to participate [14].

### Barriers and enablers in the recruitment and participation of minority ethnic groups in clinical trials

Several barriers and enablers influencing the trial recruitment of people from minority ethnic groups were identified. Interestingly, certain factors could act as both a barrier and an enabler depending on how they were applied.

Issues with communication acted as a barrier in the recruitment of patients from minority ethnic groups, particularly in relation to patients who spoke different languages to those spoken by staff, or when patients had a low level of understanding of research designs.

Key enablers identified included the willingness of researchers to establish compelling associations with communities [33] and ensuring representation of minority ethnic groups within the research and healthcare staff [17]. Additional factors acting as barriers and enablers are described in Table 2.

**Table 2:** Factors acting as barriers and enablers discussed in articles

Categories	Barriers	Enablers
Communication	– Language barriers and a lack of understanding of what it means to participate in a research project. These difficulties were linked to significantly fewer Hispanic patients being included in acute settings [15] – During the early stages of recruitment consent forms, assessments and procedures were long, which dissuaded participants to join [34] – The absence of a workable method to translate research materials like quality-of-life measurement tools and informed consent documents [36] – It was challenging to adopt techniques (e.g. use of interpreters) in primary care settings due to everyday care and busy services [16]	– Involving the target population and stakeholders in the design and evaluation of health education materials contributed to increasing scientific accuracy, reducing complexity, achieving appropriate readability levels and ensuring cultural appropriateness [48] – Researchers can also make their study more attractive to minority ethnic groups by highlighting how their study will benefit specific communities [57]
Staff interactions	– The way healthcare providers interact with African American cancer patients results in both trust and mistrust in the healthcare system. A positive experience fosters trust, while a negative experience fosters mistrust [43] – Poor patient-provider interactions contributed to distrust in the healthcare system and lower participation in clinical trials [43] – Staff must understand that they are not exempt from racism. They need to recognise their own values and beliefs towards minority ethnic groups, and how this might affect clinical trial recruitment [43]	– Patients’ willingness to take part, continue with the intervention and openness to taking part in subsequent clinical studies were all significantly influenced by the study team’s interaction [33] – One of the most successful retention techniques for clinical trials targeting African Americans was for the principal investigators and study coordinators to build rapport with participants [33] – Study staff, doctors and nurse practitioners’ willingness to interact with patients, attend to their individual cardiovascular health metrics and get in touch with their private physicians [33] – The study team’s steadfast presence and excitement, as well as their readiness to work out with the participants. Many people claimed that this had a significant and uplifting effect on them [33] – Researchers who connect with the community before recruiting for research studies establish a trusting relationship with participants [10, 17] – Food played a significant role in recruitment. Several participants mentioned that they went because food was provided, which helped to improve attendance [11, 17] – The key to a successful recruitment effort ultimately turned out to be perseverance in the search for locations that permitted food service. By demonstrating that the researchers view the participants as people rather than as research subjects, providing culturally and nutritionally appropriate food that does not exacerbate health disparities [11] – Long-term connections with communities may also enable researchers to implement new recruitment tactics as needed. Being trustworthy continues to be important for both research participation and recruitment [33, 51]
Representation	– Minority ethnic communities continue to be dubious of the intentions and objectives of academics, particularly when those researchers are all White [11]	– Participants attributed their initial willingness to sign up for the intervention to having a team of researchers that “looked like me” or due to “the doctor being Black”. A participant felt better “understood” by the programme researchers, as they could better relate to his challenges as a Black man in the US [33, 43] – Interacting with African American research staff who are well-known in the community and share a common identity (e.g. cultural norms, experiences, background) fosters trust [17] – Increasing diversity among professional stakeholders and patient populations at respective centres would also likely improve understanding across cultural frameworks and potentially mitigate bias [53]
Socioeconomic factors	– A health system barrier to appropriate healthcare, including participation in clinical trials, was the absence of appropriate medical insurance [41, 43, 57] – Minority ethnic group members are discouraged from participating in studies due to mistrust, concerns about receiving poor-quality medical care and risks to insurance coverage or legal status [57] – Lack of patient availability due to competing demands, restricted knowledge of clinical trials, stringent inclusion or exclusion criteria, uncertainty about residency status, fear of harm and lack of insurance coverage for treatment [11, 43, 46, 58, 60, 61, 63] – Different types of trials such as personalised trials (N-of-1) reported that participation was limited due to lack of insurance [14] – Minority ethnic groups have problems accessing timely treatment options due to psychological factors that might be triggered by elements such as socioeconomic class or cultural phenomenon, which impact clinical research enrolment [58]	– Patients with social support and mental health services available to them, even if they may be less inclined to request them, are an important technique to enhance study enrolment. Making these services accessible and letting patients know about them during clinic visits may boost usage in the future [10, 43, 45, 46] – Considering both potential financial difficulties and participant comfort levels, the groups needed to meet in suitable places. Incentives (mostly monetary) are potential recruiting facilitators that should be used to emphasise that participants’ time and opinions are significant [11, 33, 37, 47, 54, 65]
Study characteristics	– Inadequate funding for clinical trial efforts in regions that assist patients of minority ethnic groups [32] – The pressure of the study requirements and mistrust of the research method were major inhibitors [45] – When resources such as electronic health records have inaccurate or missing race-ethnicity fields, it was difficult to identify prospective participants from minority ethnic groups [46] – Participation barriers seem to be more common during the early stages of recruitment [34] – Strategies to advertise in Black and Hispanic communities were based on funding allocation, which tends to be minimal for relevant expenses such as transport or accommodation [53] – The use of lengthy consent forms with both medical and legal jargon makes comprehension more difficult [11, 38]	– The role of the family in recruitment and participation, the interest in education as a main characteristic in trial design, and the desire for “lifestyle” as a treatment option [14] – Community partners provide perspectives on their community’s needs, interests and concerns, as well as help with study design, communications and recruitment strategies [57] – Reliance on community relationships, including those with churches and other local organisations, was common in recruitment efforts. All the barbershops that cut the hair of the research team members were open to exchanging information and talking about joint ventures, emphasising the value of fostering relationships to reduce Black communities’ fear of doctors [11] – When trials were suggested by physicians or conducted in community-engaged institutions, without industry funding, participants were more likely to engage [63] – Participants’ trust was higher in university hospitals and general practitioners. Latin-American participants had a higher percentage of trust in pharmaceutical companies in comparison to African-American participants, regardless of whether they were from rural or urban settings [12] – Nationwide registries where volunteers received information and invitation about studies, they might qualify for are a crucial tactic for boosting minority groups’ enrolment [9]

## Discussion

The present systematic review revealed gaps in the available literature and limited generalisability of findings. The evidence included in the review was of moderate quality (in average). Nevertheless, it highlighted potential ways to address the underrepresentation of minority ethnic groups in clinical trials.

Predominantly, most of the studies reviewed originated from the US, with African American and Black patients being the main participant population investigated. The focus on oncology further narrowed the range [71–73].

There was little examination of barriers to the recruitment of other US minority ethnic groups, such as Korean Americans, Hispanics and Latin Americans. Given the cultural and experiential specificities rooted in the US, these findings are unlikely to be readily transferred and applied in other countries [57, 74]. Only seven studies were based in other countries, with just one study conducted in a country catalogued as a low- to middle-income country (LMIC) [56].

In addition, only a few studies investigated the reasons behind withdrawals from clinical trials. It is important to address these clear gaps in the literature and encouraging future research on trial recruitment of minority ethnic groups outside the US and the factors prompting withdrawal from trials. The systematic collection of data on ethnicity, robust reporting and widely available published findings would be essential in achieving this.

A previous report by Skyers et al. [75] from the Basil Skyers Myeloma Foundation has also underlined gaps, particularly on the disproportional impact of blood cancers on Black communities in the UK. The report urged for collective efforts from research organisations, funding institutions and providers to investigate further on the matter, considering interactions such as social location, language, class and gender [75].

Recruitment challenges are multifaceted, and findings may not always indicate an immediate solution. For example, while trial location may inadvertently exclude some populations from participation, in some instances, these populations may not engage these healthcare services altogether or experience poor care and disengage and thus are inaccessible to researchers irrespective of location [52]. Although some exclusions might be beyond the researcher’s control, other aspects, such as research design, could be anticipated and managed [34, 52, 57].

Several recommendations have emerged from the present review to enable more patients from minority ethnic groups to be recruited to trials. However, their practical implementation might still face additional challenges.

For instance, the introduction of diversity quotas for trials may result in recruitment taking longer and increase the cost of delivering the study, ultimately reducing the benefit of the research to all patients [76]. Other impediments could be the cost and time needed to provide quality translations and interpreters, and nuances with the accuracy of translations, especially for cognitive scales questions [44].

A more flexible approach, such as using the INCLUDE Ethnicity Framework [77], may strike a more favourable balance between achieving diversity of trial recruits and producing timely results. This framework allows research teams to be reflective in their recruitment practices regarding who they are approaching and how the research design may impact on a patient’s ability to access and take part in a trial [77].

Similarly, other studies have reported that Patient and Public Involvement and Engagement (PPIE) have shown a positive impact on diverse recruitment. For instance, Rayment et al. showed that engaging women through PPIE practices increased their awareness of both the potential benefits and risks of using probiotics during pregnancy [78].

Regarding the recruitment of Black and African American patients, the reviewed studies produced a significant amount of data on how historical and institutional forms of racism acted as barriers to trial participation [9–11, 37, 53, 55], exemplified by events like the US Tuskegee Syphilis study and its unethical treatment of Black patients [74]. The Tuskegee Syphilis study was mentioned in 6 of the 36 papers from the US, all of which were from 1st January 2017 to 1st October 2022.

The findings indicated that patients’ trust was gained when the research team were of the same ethnicity and demonstrated cultural competence, possessing an awareness of the patients’ culture and experiences [17]. As a result, it was recommended that research teams include minority ethnic representatives [32] and culturally sensitive staff on their teams [9].

Further trust building could be achieved through community-based [11, 57, 60, 79] and participatory approaches to research. In this approach, there is a focus on building community relationships prior to initiating research, and encompassing community members in the practice of research, including study design, recruitment and communication approaches. This has shown to, not only facilitate trust building, but also to highlight to the community the potential benefits of a trial and patient preference to researchers [57]. Community approaches could also enable the use of trusted venues that are easily accessible both financially and geographically, such as churches and barbershops [10, 40].

The Yale School of Medicine’s initiative of leveraging cultural ambassadors exemplifies such a strategy [80]. These cultural ambassadors acted as the bridge between the medical school and communities, supporting the development of protocols, recruitment plans, translation services and planning community engagement activities [80]. These activities could also bridge the gap for researchers where patients do not present to the clinic and, consequently, are not invited to trials. Nevertheless, community-trusted settings might not be suitable for all types of trials, since more complex interventional clinical trials would require purposively equipped settings for recruitment.

The present review highlighted a scarcity of studies focusing on the reasons behind patients’ withdrawal from clinical trials, emphasising that the retention of trial participants is as important as their recruitment. While some measures have been suggested to improve retention this area, they remain notably under-researched [78].

From the limited information available, it appeared that some reasons for ethnic minority participants leaving a trial early mirror the reasons some never participated in a trial in the first place. These included time and schedule limitations, language barriers and immigration status. It would, therefore, be beneficial to research trial retention alongside recruitment uptake. For example, Kearney et al. indicated that protecting against attrition should begin at the recruitment phase, with provision of transparent information on the withdrawal process and employing flexible data collection methods, such as using routine appointments or medical notes to reduce patient burden [79]. Similarly, Otado et al. highlighted addressing participant concerns as one of the most reported strategies to aid in retention [79].

The present review also underscored the value of patient navigators [41], community liaisons [40] and cultural ambassadors [80] in the recruitment of patients from minority ethnic groups into clinical trials. These supportive roles not only carried out recruitment, and explained the research process [40, 41], but also provided emotional support [45] and were considered as peers and relatable Figs. [40]. Kearney et al. further proposed that patient navigators could engage with participants contemplating leaving the trial, to explain the withdrawal process along with any alternative data collection options [79].

In further discussions about the role of patient navigators, feedback from patients recommended that navigators should have a good working knowledge of the trial process, and soft skills such as being compassionate, and ability to bridge the communication gap between patients and researchers [45].

Lastly, some studies highlighted that the required interventions might surpass the capabilities of participants and researchers alone, and stressed that funders, policy and public health bodies should work in partnership to cater to the broader public’s needs, and not just parts of it [3, 24, 71]. For example, barriers related to socioeconomic status and access to care and socio-political factors, such as discriminatory policies [5], need to be addressed by robust societal and healthcare policies [72].

### Strengths and limitations of the review

The robustness of the present review was strengthened by having two reviewers during the screening phases, ensuring the accuracy and the relevance of the peer-reviewed articles, and performing a collaborative quality assessment. In addition, feedback from stakeholders at Roche and UCLH was incorporated throughout the review process. Contributions from patient representatives played a pivotal role, ensuring that the review remained grounded in-patient experiences and perspectives.

The present review followed a robust methodology, with more than 18,000 records of peer-reviewed journals screened. Nevertheless, despite utilising a well-tested search strategy across four distinct databases, it is inevitable that some useful articles might have been overlooked. This could be attributed to the specifics of the inclusion criteria, or nuances in keywords coding. It is acknowledged that by making commentary on enablers and barriers as part of our inclusion criteria, a proportion of articles relevant to the topic at hand might have been excluded.

The scope of the present review was limited to empirical articles, and as such, non-empirical articles or grey literature were excluded. Such literature could have provided additional experiences and reflections on clinical trial disparities. Furthermore, this review did not include paediatric studies, where enablers and barriers of trial participation might differ considerably from those of adults [80].

Lastly, article quality was critically appraised using the MMAT. The overall quality of the articles reviewed was of moderate quality; with 21 studies scoring above 3, showcasing their robustness. However, there were 12 articles with scores of 3 or below. Their main limitations included lack of detail in reporting, incomplete data collection and sampling bias.

## Conclusion

Despite strides made towards enhancing clinical trial diversity, there remains a substantial gap to better understand and address the reasons for underrepresentation, particularly in regions outside the US that lack comprehensive data on this issue. Various barriers, such as limited access to healthcare, challenges in transportation, constraints in taking time off work and existing cultural and linguistic impediments, continue to hinder diverse participation in clinical trials. Addressing these challenges requires greater efforts to engage and include individuals from minority ethnic groups in clinical trials, to ensure all patient populations have equal access to the benefits of medical research. The present review has highlighted the significance of reaching out to and educating underrepresented communities, providing information in a culturally sensitive and accessible manner, and actively engaging with community leaders and organisations to build trust and encourage participation. Lastly, it is essential to recognise that addressing these changes would require appropriate discussions and planning to secure the necessary funds and resources and for all roles involved in the design and delivery of research to begin their own learning journey around bias, allyship and anti-racism.
